# How AI agents search

**DOI:** 10.1371/journal.pone.0360157

**Published:** 2026-10-21

**Authors:** Francesca Pezzuti, Ophir Frieder, Fabrizio Silvestri, Sean MacAvaney, Nicola Tonellotto

**Affiliations:** 1 Department of Information Engineering, University of Pisa, Pisa, Italy; 2 Department of Computer Science, Georgetown University, Washington, District of Columbia, United States of America; 3 Department of Biostatistics, Bioinformatics, and Biomathematics, Georgetown University Medical Center, Washington, District of Columbia, United States of America; 4 Department of Computer, Control and Management Engineering, Sapienza University of Rome, Rome, Italy; 5 School of Computing Science, University of Glasgow, Glasgow, United Kingdom; Universidad Diego Portales, CHILE

## Abstract

Large Language Models (LLMs) have evolved from relying primarily on knowledge stored in their parametric memory to using non-parametric memory mechanisms such as Retrieval-Augmented Generation (RAG) to provide and ground knowledge. Recently, LLMs have begun to perform retrieval autonomously within a reasoning loop, an approach referred to as Agentic RAG. This shift introduces new querying behaviours, with implications for the design of retrieval systems that have yet to be evaluated. In this work, we introduce a large-scale dataset (ASQ) capturing how Agentic RAG systems perform multi-step search and reasoning. ASQ contains about 1M agentic search traces collected from open-source Agentic RAG systems over 3 corpora, 5 agent variants, and 6 retrieval settings. Our analysis of ASQ shows that, within this controlled benchmark setting, a single search query can trigger long chains of retrieval calls (up to 186) and follow query reformulation dynamics different from those observed in historical human-search data. Our results also reveal substantial behavioural variation across agent and retrieval configurations: changes in re-ranking and retrieval depth do not consistently affect search workloads in terms of search-call volume and trace length across agents. To our knowledge, publicly available production logs do not expose the agent, dataset, and retrieval configuration associated with each trace. ASQ provides a reusable resource for further analysing agent search behaviour and supporting future information retrieval studies on agentic workloads under controlled settings.

## Introduction

Search systems are increasingly used by both human users and autonomous AI agents, a shift largely driven by the introduction of Large Language Models (LLMs) [1,2]. Originally, LLMs were designed to answer questions by relying on knowledge acquired during training, which is stored in their parameters [3,4]. However, to improve answer factuality, broaden coverage, and provide LLMs with access to up-to-date and external information during response generation, they are now frequently combined with retrieval components [4,5]. This approach was popularised by the Retrieval-Augmented Generation (RAG) paradigm [4,5], in which the system retrieves relevant information from an external knowledge source once before generating an answer.

More recently, RAG has broadened into agentic RAG [6–8], in which the system autonomously decides when, what, and how to search, iteratively refining its queries based on intermediate results to tackle complex questions with more grounded and accurate answers. This advance, however, has not only improved the quality of the final answers produced, but also changed the way search is carried out. For instance, agents can generate many intermediate queries, revisit earlier ones, and adapt their search strategy dynamically as they gather new knowledge [6,7].

Retrieval systems are therefore increasingly exposed to two streams of queries: those generated by humans (*organic query stream*) and those generated by AI agents [1,2] (*synthetic query stream*). Historically, however, search systems were developed to serve human users [9]. As a consequence, most retrieval methods, benchmarks, and evaluation practices are designed around their *search behaviours*. In particular, prior research has extensively studied *session-level search* [10–14], i.e., how humans search with sequences of queries while trying to satisfy an information need or complete a task, using behavioural data collected from human users such as query logs [13,15] and shared evaluation tasks [16–18]. These studies informed the design of search system components such as query suggestion [19], personalisation [20,21], caching strategies [22], and other forms of retrieval optimisation [23].

As retrieval systems increasingly serve both humans and agents, it becomes important to understand whether these two query streams place similar demands on them. Existing evidence suggests that they may not. First, human search sessions have been observed to contain approximately two searches on average [14]. In agentic RAG systems, and more broadly in tool-using agents, query formulation can be part of an automated retrieval and reasoning loop in which the system may generate an initial query, inspect retrieved evidence, and then issue reformulated or follow-up queries as part of the same task [24,25]. This may increase both the number and rate of retrieval requests associated with a single task, since agentic reformulation is automated rather than constrained by the time humans need to inspect results, type new queries, and decide how to reformulate them. Second, human-generated traffic typically follows seasonal and diurnal patterns [13,14], partly reflecting users’ daily activity cycles. Continuously operating agents may produce different temporal workload patterns, for example when automated tasks are executed independently of individual users’ activity cycles [2,26]. One possible implication is that predictable low-traffic periods for maintenance or resource allocation may become less pronounced. However, the practical impact of such patterns remains deployment-dependent. Third, queries generated within LLM-based agents may differ from human-written queries in style and structure, for example in length, syntactic form, specificity, and vocabulary [27]. Since retrieval systems can be sensitive to query formulation [28], these differences may influence lexical matching, semantic similarity estimates, and therefore could also affect which documents are retrieved and how they are ranked. Overall, these three differences may matter for retrieval engines because they affect query content, retrieval frequency, request timing, and the interaction between query formulation and ranking. These workload properties, in turn, raise questions about whether the demands placed on retrieval systems in terms of efficiency, effectiveness, robustness, and evaluation may have changed. More broadly, as AI agents become embedded in education [29], healthcare [24], scientific [30], commercial [31], e-Governance [32,33], and everyday applications [34], understanding how they search becomes critical to ensuring that these systems operate reliably, can be rigorously evaluated, and can be trusted.

Despite the growing use of agents, we still lack step-by-step data showing how they search in practice. In particular, there is currently limited reusable data capturing how AI agents reformulate queries and retrieve information across multiple steps. As a result, it remains unclear whether retrieval methods developed for human-driven search remain suitable for agents, or how they can be optimised to support it effectively.

To address this gap, we propose a methodology for building datasets that capture the search behaviours of AI agents in a controlled setting, as existing logs of agent-generated queries do not jointly provide the complete agent, task, and trace context required for this analysis. Specifically, we propose to log the intermediate synthetic queries, retrieved documents, and reasoning descriptions produced by open-source AI agents to guide follow-up searches and the generation of final answers. Using this methodology, we construct the Agentic Search Queryset dataset (ASQ), a large-scale dataset of agentic search traces from open-source agents. Since the landscape of agentic systems is evolving rapidly, we recognise that ASQ may become outdated over time, as newer agents may exhibit search behaviours that differ substantially from those captured in ASQ because they are trained differently or operate with different action spaces. We therefore also release on GitHub the code for extending ASQ with future agents and future retrieval systems.

Our ASQ dataset enables the study of how agents interact with retrieval systems over time, including session-level search behaviours such as query reformulation and decomposition, variation in the number of search steps required to answer, and failure cases such as repeated or unproductive queries, unsuccessful decomposition strategies, and cases in which retrieved documents do not provide enough support for a correct final answer. As far as we are aware, production logs of user queries submitted to agents are not yet publicly available. We therefore ground ASQ in three established benchmarks with document relevance labels: HotpotQA (test) [35], Researchy Questions [36], and MS MARCO dev [37]. With ground-truth answers also available for HotpotQA, ASQ can support the analysis of agent search behaviour, answer quality, and the evaluation of retrieval methods in agent-driven settings.

Benchmark-derived queries may differ from production query logs, but they provide relevance judgements, reproducible inputs, and controlled comparisons across agents and retrieval configurations. The same reproducibility considerations also shape the agent selection. We focus on open-source agents rather than commercial systems, whose undocumented updates or removal can make results difficult to reproduce consistently. This focus is further constrained by the lack of comparable trace-level access in proprietary systems. However, behaviours observed in open-source agents may not fully generalise to proprietary systems. Within this controlled setting, we can reproducibly compare observable trace properties of agentic RAG systems across agents, retrieval pipelines, and retrieval depths, while leaving production agent traffic and temporal workload dynamics for future work.

Finally, since inference with agentic RAG systems is computationally expensive and time-consuming, releasing ASQ contributes to more accessible, reproducible, and resource-efficient research. Overall, ASQ spans 3 input corpora, 5 agent variants, 2 retrieval pipelines, and up to 3 retrieval depths, comprising a total of 1.02M traces. We use these traces to analyse agent search behaviour from a retrieval-system perspective, focusing on observable trace properties such as search-call volume, trace length, intermediate-query length, and reformulation patterns, while discussing their possible implications for retrieval systems.

**Research questions** The main contribution of this work is the construction of ASQ, a dataset of complete agentic search traces collected using multiple open-source agents and retrieval systems across several query sets and retrieval configurations. We release ASQ as a reusable resource for studying agent search behaviour. In this work, we provide an initial analysis of the dataset and illustrate the types of questions it can support by addressing the following research questions:

- **RQ1:** What search-call volumes, trace lengths, and intermediate-query lengths are exhibited by the open-source agents under study across query sets and retrieval settings?
- **RQ2:** How does trace length vary for matched input queries when re-ranking is introduced or retrieval depth is increased within the evaluated agent-dataset configurations?
- **RQ3:** What similarities and differences are observed between the search patterns in ASQ and those reported for human sessions in historical search-engine query logs?
- **RQ4:** How do the observed agent query-reformulation patterns evolve across successive trace iterations?

To further illustrate how ASQ can support analyses connecting trace behaviour with downstream outcomes, we also examine the following auxiliary research question on HQA, for which ground-truth answers are available:

- **AQ1:** How are trace length and within-trace query repetition associated with final-answer correctness?

These questions provide an initial characterisation of ASQ, while the released traces can support further analyses and evaluation protocols beyond those considered in this work.

## Related work

This section reviews prior work most relevant to our study, focusing on how human search behaviour has been analysed in information retrieval and on recent advances in agentic RAG systems.

### Human search behaviour

Human search behaviour has been studied extensively in Information Retrieval (IR), and progress in the field has long relied on realistic data derived from human search activity [9]. These resources have supported the study of how people reformulate queries, browse results, and pursue information over multiple search steps, and have helped shape the optimisation and evaluation of retrieval systems, user models, and IR metrics.

In particular, the IR community has often sourced organic queries from active search engines. For example, the TREC-8 Large Web Track sourced queries from AltaVista, a popular commercial search engine at the time. More recently, popular datasets such as MS MARCO [37] and Natural Questions [38] have sourced their queries from Microsoft Bing and Google, respectively. On a few occasions, search engine query logs containing session-level information, such as the sequences of queries submitted by the same user, their timestamps, and clicked links have been made available [13,15]. These logs support the study of real-world query distributions, temporal patterns, user heterogeneity, and large-scale session behaviours, but their use in research has been limited by ethical and legal considerations. Finally, controlled evaluation resources, such as those provided by TREC [16–18] or UQV100 [39], offer alternative ways to study session-level, conversational, and interactive search behaviours. However, concerns about how realistically these resources reflect real search behaviour have long been present in IR research [40].

However, these human-centred datasets are not suitable for studying AI agents. Given IR’s long-standing reliance on user behavioural data, designing and evaluating retrieval systems for agent use is challenging without datasets that capture how agents issue intermediate queries, revisit earlier searches, and adapt their strategies across multiple retrieval steps. To fill this gap, we develop a methodology to build datasets that capture how RAG agents search. Using this methodology, we release ASQ, a dataset capturing a fine-grained view of how these systems perform multi-step search.

### Agentic RAG

*Retrieval-Augmented Generation* systems (RAG) generate responses using information retrieved from external sources [4,5], which helps overcome the limitations of relying only on knowledge stored in model parameters [4]. However, these RAG systems may struggle with complex or unfamiliar questions that require more than one retrieval step [7,41], namely multi-faceted or multi-hop queries.

To address this, recent work has introduced *agentic RAG* systems, or *agents* for short, such as as Search-R1 [42], AutoRefine [43], and related approaches [42,44]. These systems answer questions relying on a multi-step process, which we refer to as a *reasoning loop*, in which reasoning [45], retrieval, and answer generation inform one another. During this loop, which terminates upon answer generation or encountering run-time errors, agents reason over their internal knowledge, the retrieved documents, and issue follow-up, refined queries [41,45]. During this process, they decide when and what to search for while coordinating reasoning, retrieval, and answering.

Agentic RAG systems exploit different coordination mechanisms to implement this multi-step process. Some open-source agents coordinate actions by generating specialised XML-style control tags, as Search-R1 and AutoRefine [42,43]. Tool-using agents instead invoke retrieval through external tools, search APIs, or function calls [25]; to do so, they typically invoke a retrieval function through a structured interface, passing the search query as an argument. Proprietary systems, such as Google Generative Search [46], GPT search [47] and others [48,49], are also relevant deployed examples of agentic search, but their internal coordination mechanisms and trace-level data are typically not exposed. At the same time, industry reports indicate growing use of open-source and open-weight models, especially in deployments where privacy, customisation, and infrastructure requirements are important [50,51].

The next subsection reviews recent efforts to analyse agent search behaviour using real-world logs, controlled task settings, and intermediate retrieval signals.

### Agent search behaviour

Although substantial progress has been made in the design and evaluation of agentic RAG systems [25,42,44,52], their search behaviour remains comparatively underexamined. Researchers are beginning to address this problem through real-world traffic analysis and controlled benchmarking.

In a study contemporaneous with ours, Ning et al. [53] release and analyse a real-world, raw search log of about 14 million queries sourced from a search API accessed by external agentic clients. Their study examines query frequency, geographic search request provenance, session dynamics, and cross-step evidence reuse. However, the logs do not expose the original user task or prompt, the client-side model and control policy, the agent’s memory state, or how retrieved evidence was presented to the agent. They therefore do not permit controlled attribution of behavioural differences to particular agent designs or direct comparisons across known agent configurations.

A related study examines agentic search under controlled but realistic task conditions. He et al. introduce LocalSearchBench [54], a benchmark built from real user queries in the local-life services domain and an interactive environment in which RAG agents retrieve information about merchants and products through multiple tools. The study evaluates end-task correctness, completeness, and faithfulness under known task and environment conditions. However, its primary focus is on task completion within a specific vertical domain rather than retrieval-system-facing querying behaviour.

Tian et al. [55] study intermediate queries produced by Search-R1 [42] and R1-Searcher [52] using query-performance prediction signals. Their results show that more effective retrievers can improve answer quality while reducing the number of searches, and that query-performance estimates are positively associated with final-answer quality.

Whereas prior studies primarily examine deployed traffic, domain-specific task performance, or the relationship between retrieval quality and answer accuracy, our ASQ dataset focuses on the observable query process. It captures the intermediate queries, retrieved documents, reasoning descriptions, and final answer associated with each initial query under known experimental conditions. This supports reproducible analysis of how agents reformulate, repeat, and terminate searches, as well as comparisons across retrieval and agent configurations.

## Terminology

Given an initial query *q*_0_, we define an agent’s attempt to answer it as an agentic run, or *arun* for short, i.e., the sequence of actions performed by the agent to answer *q*_0_. Actions can be operative, such as query formulation, retrieval, and answer generation, or reflective, such as reasoning and information refinement, which produce natural-language descriptions that guide subsequent behaviour. Not all aruns necessarily yield a final answer: they may terminate prematurely due to run-time errors, or an explicit early stop. Unlike *human search sessions*, which comprise the queries submitted by a user within a time window and may include parallel searches across multiple tabs [56], aruns are bound to a single initial query *q*_0_.

We now define two data abstractions needed to describe our data collection methodology: *frames* and *traces*.

A frame *f* records the outputs produced by the *actions* the agent performed during a specific iteration of an arun:


$$\begin{array}{c} f=(q,{ℛ}_{q},𝒟) \end{array}$$

where *q* denotes an intermediate query generated by the agent, ${ℛ}_{q}$ is the corresponding ranked list of documents retrieved from the corpus, and 𝒟 is the list of descriptions produced by agent-specific reflective operations.

Given an initial query *q*_0_, we define a *trace* as the ordered collection of frames belonging to the same arun started with *q*_0_ and paired with the answer *a* generated by an agent *A*:


$$\begin{array}{c} {𝒯}_{A}(q_{0})=(S,a)\;\text{with}\;S=(f_{1},...,f_{N}), \end{array}$$

where $f_{i}$ is the frame associated with the *i*-th iteration of the arun. The sequence *S* contains *N* frames, and we refer to *N* as the *trace length*. If the agent answers *q*_0_ directly without issuing any retrieval request, then *N* = 0 and *S* is the empty sequence.

It is worth mentioning that the answer can be empty if no answer is returned due to early exit or errors. We define the traces of aruns with no answer as *incomplete*.

While a frame captures the outputs produced in a single iteration, a trace is a representation of an arun capturing all the intermediate and final data an agent produced. Frames encode the temporal and causal dependencies between an agent’s actions; thus, collecting traces enables analysis of action-level interactions and the agent’s search session behaviours.

## Dataset construction

We define the characteristics that our dataset must satisfy to capture agents’ step-by-step search behaviour, and then present the methodology used to collect agentic traces and construct ASQ.

### Properties

We now outline the *intrinsic* and *extrinsic* properties of our dataset, i.e., the characteristics that our dataset should have independently of any use case, and those enabling downstream uses, respectively.

**Intrinsic** Each query should be unambiguously linked to its corresponding trace, answer, and all associated frames, and vice versa (*traceability*), with the temporal sequence of actions — both within and across frames — fully reconstructable (*chronological order*). Each frame should capture all data produced during its iteration, and each trace should include all its constituent frames (*completeness*). Moreover, the *integrity* of agent behaviour must be preserved: logged data should remain raw and unaltered, the agent should not be prompted to decompose or reformulate queries, and no interventions that could influence its decision-making process should be introduced. Finally, the dataset should ensure *diversity* across agents and retriever configurations, query types, corpora, and domains.

**Extrinsic** The dataset should support both the optimisation and the rigorous evaluation of retrieval models and systems (*optimisability* and *assessability*), enabling systematic comparison across approaches and configurations. In addition, the methodology should be compatible with a variety of agents and retrieval systems (*interoperability*), allowing different model architectures and implementations to be seamlessly integrated. Finally, both the dataset and the methodology should guarantee *extensibility*, so that new agents, retrievers, tasks, or evaluation protocols can be incorporated over time without requiring structural changes.

### Methodology

We now describe our dataset collection methodology, designed to systematically log agentic RAG systems’ search behaviours.

#### Prompt construction and agentic run start.

To initiate the arun, we provide the agent with a prompt that incorporates the organic query according to each agent’s specifics.

#### Iterative extraction of frames.

During each arun execution, all trace data are extracted during generation rather than after the entire sequence has been generated. This enables logging incomplete aruns, ensuring *integrity*. As mentioned, some agentic RAG systems coordinate their actions by generating specialised tags, which may vary across agents. To collect frames across these settings, we parse tag-based retrieval actions using regular expressions and intercept retrieval tool calls for tool-calling agents. In both cases, for each retrieval action we then record the identifiers of the retrieved documents, preserving storage efficiency. While in the remainder of this section we refer to the tags used by Search-R1 and AutoRefine, the approach generalises to other agents with other control tags. For tag-based agents, each time the agent emits a synthetic query within <search> tags, we extract it from tags and store it. We then log the identifiers of the retrieved documents by intercepting the retriever’s returned results before their content is wrapped within <information> tags and fed again to the agent.

Similarly, thoughts, i.e., natural-language descriptions generated during chain-of-thought reasoning [45], are extracted from <think> tags for tag-based agents. For tool-calling agents, we instead record the reasoning field exposed by the agent interface. For tag-based agents implementing an explicit refinement phase (e.g., Autorefine), during which the agent extracts and organises information from the retrieved documents, the output of the refinement step is extracted from <refine> tags [43]. Finally, we extract the final answer from <answer> tags for tag-based agents, and from the final message for tool-calling agents. The extraction of the final answer signals the successful termination of the agentic arun.

#### Run-time error handling.

Data extraction order is model-specific; incorrect ordering may trigger regex failures, so practitioners should follow each model’s specifics. Moreover, tag-based agents may generate spurious or unexpected text, triggering regex failures, and similarly, tool-calling agents may issue missing, malformed, or failed tool calls. In both cases, when an action’s parsing fails, we terminate the arun early and retain it as incomplete. By avoiding post-hoc trace repair, we preserve data integrity. Finally, we consider legitimate, and log, empty retrievals where no documents are retrieved.

#### Post-processing and storage.

To ensure *integrity*, we do not apply any post-processing or filtering, and we retain incomplete aruns. We store traces in a structured format, with each frame and its associated data kept separately to preserve *traceability*, *chronological order*, and selective access. Data formats are extensively described in the next section.

## Dataset

We now describe the structure of ASQ, including how the data are organised, stored, and made available for use. We publicly release ASQ at:

- https://huggingface.co/datasets/AgenticSearchQueryset/ASQ.

### Format and organisation

Each frame is assigned an increasing iteration identifier corresponding to its position within the trace, ensuring *traceability*. For the agents considered, which perform one action per type per iteration, a *chronological order* is inherently preserved.

We store each trace in its own directory, with logged data shared by data type (queries, ranked lists, etc.) and stored in separate tsv files. This design allows users to selectively access only the traces or artefacts relevant to their experiments without downloading the entire dataset.

Each trace comprises four tsv artefact files, with rows associated with a qid corresponding to the original query:

- *answers*: qid (str), answer (str)
- *synthetic queries*: qid (str), iteration (int), llm_query (str)
- *thoughts*: qid (str), iteration (int), thought (str)
- *ranked lists*: qid (str), iteration (int), docid (int), rank (int)

### Ethics statement

ASQ is derived from publicly available datasets and intended solely for research on agentic search behaviour. The authors do not endorse or assume responsibility for the content or biases in the traces, which do not represent the views of the researchers or their institutions. Users are advised to apply appropriate safety and content filters. No human subjects were involved, and no human evaluation of agent outputs was conducted as part of this study.

## Experimental setup

We now describe the experimental setup used to build our dataset.

### Agents

We use Search-R1 [42] and AutoRefine [43] as search agents that generate explicit XML-style control tags, and GPT-OSS [57] as a tool-using agent. We exclude R1-Searcher [52] as it is outdated, and more advanced agents such as ParallelSearch [44] because they are not publicly available yet. Commercial and proprietary agents are also excluded, as they do not expose the intermediate data required for query tracing. For Search-R1 we use generator models of varying capacities, optimised with exact match and PPO, whereas AutoRefine is only available with the Qwen2.5-3B-Base generator. Finally, for GPT-OSS we use the gpt-oss-20b generator, an open-weight reasoning model post-trained to natively support reasoning and tool use before producing an answer [57]. In our setup, this support is used to invoke the retriever through its structured tool-call interface, rather than using XML-like control tags. Table 1 summarises the agent variants and the checkpoints used in our experiments. When generating traces, we use standard stochastic decoding with a temperature of 0.7.

**Table 1:** Agent checkpoints and variants used in our experiments.

Agent	Variant	Checkpoint
Search-R1	Qwen-3B	PeterJinGo/SearchR1-nq_hotpotqa_train-qwen2.5-3b-em-ppo-v0.3
	Qwen-7B	PeterJinGo/SearchR1-nq_hotpotqa_train-qwen2.5-7b
	Qwen-14B	PeterJinGo/SearchR1-nq_hotpotqa_train-qwen2.5-14b-it-em-ppo-v0.2
AutoRefine	Autorefine	yrshi/AutoRefine-Qwen2.5-3B-Instruct
GPT-OSS	GPT-20B	openai/gpt-oss-20b

Unless otherwise stated, we set the number of retrieved documents fed to agents’ generators to the commonly used *k* = 3 [58]; on HQA, we evaluate k∈{3,5,10} to study the effect of retrieval depth on search behaviour. For the retrieval pipeline, we use two configurations: BM25 [9] (a standard lexical retrieval method) and BM25 followed by MonoElectra cross-encoder re-ranking [59], which refines the initial top-100 BM25 ranking using a more accurate neural model.

### Input datasets

We rely on benchmark-derived queries as input to the agents, which may not reflect real-world agent production traffic. To our knowledge, however, search logs of user queries submitted to agents are not available. Benchmark-derived queries therefore provide a controlled substitute: their task settings and query types are documented, their corpora are known, and they are often paired with relevance judgements or ground-truth answers.

To support *optimisability* and *assessability*, we ground our dataset on IR datasets for which relevance judgements are available. To ensure *diversity*, we generated traces for informational queries of varying types: factoid queries from MS MARCO dev (MSM) [37], multi-faceted queries from HQA (test split), and open-ended researchy questions from RQ [36]. Note that since some of the agents under study have been fine-tuned on Natural Questions [38] and HQA (train split), we do not generate traces for their queries. We also exclude BRIGHT [60] as its tasks are highly domain-specific and knowledge-intensive. Table 2 summarises the main characteristics of the input datasets used.

**Table 2:** Statistics and characteristics of the organic query datasets used in the generation of the agentic query traces. Query length is reported as mean ± standard deviation in number of terms.

Dataset	Queries	Query type	Domain	Query length
HQA	7,405	multi-faceted	in-domain	15.72±5.52
MSM	101,093	factoid	out-of-domain	6.41±2.75
RQ	96,448	researchy	out-of-domain	7.79±2.86

Overall, our selection also ensures *diversity* across document types: short web passages (MSM), Wikipedia articles (HQA), and long web documents (RQ).

### Query tracing

During query tracing, we store all frames generated during each arun, ensuring *completeness*. When available, agents are provided with document titles (HQA, RQ). Following prior work [61,62], we truncate each document’s text to the first 512 tokens before passing it to the generator, in order to fit the retrieved evidence within the context. This truncation may influence agent behaviour by limiting the evidence available at each search step; however, keeping the token budget fixed ensures that the amount of retrieved text exposed to the agents is controlled across all configurations. Finally, we omit re-ranking for RQ because applying it to its substantially larger corpus would entail considerable additional computational cost.

### Reproducibility details

The code used to generate, parse, and analyse ASQ is publicly released at https://github.com/fpezzuti/ASQ. The repository includes dependency specifications, commands for building the experimental environment, and information to support reproducibility. The traces contained in ASQ were generated on a shared GPU cluster using NVIDIA A100, RTX 4090, and L40 GPUs. Since wall-clock runtime depends on the agent, query set, retrieval configuration, inference backend, batching strategy, corpus size, cluster load, and scheduling, we do not report runtime estimates. The released code supports regenerating individual ASQ subsets, allowing users to reproduce selected configurations according to their available computational resources. Given the computational cost of generating agentic traces, we generated each input–configuration once, prioritising broad coverage across configurations over repeated generation for the same setting. Consequently, this study does not quantify run-to-run variability induced by stochastic generation; repeated, multi-seed generation is left to future work. For all stochastic analysis procedures, including bootstrap resampling, we fixed the random seed to 42 to ensure the reproducibility of the reported estimates.

## ASQ analysis and discussion

In this section, we begin by reporting the coverage of our ASQ dataset and providing some trace-level statistics. We then analyse its traces from a retrieval-system perspective, examining the number of search calls, query length, and query reformulation behaviour across the evaluated agent and retrieval configurations. Finally, we compare the observed agent behaviours with those of human searchers to identify systematic differences and discuss their potential implications for retrieval systems. Together, these analyses provide initial findings from ASQ and illustrate its value as a reusable resource for studying agent search behaviour and supporting future retrieval-system evaluation.

### Dataset coverage

Table 3 summarises the scale of ASQ in terms of covered setups across corpora, search settings, *k* values, and generators, as well as the corresponding numbers of answers and search calls produced.

**Table 3:** Dataset coverage for ASQ. For each corpus, search setting, *k* value, and generator, we report the number of answers and the total number of search calls. Answer counts exclude incomplete runs and are rounded at the configuration level. Overall, including incomplete runs, ASQ comprises about 1.02M traces.

Corpus	Search setting	*k*	Generator	Answers	Search calls
HQA	Retrieval	3	Qwen-3B	7.4k	284
			Qwen-7B	7.3k	21.7k
			Autorefine	7.4k	7.4k
			GPT-20B	7.4k	37.1k
		5	Qwen-7B	7.4k	20.8k
			GPT-20B	7.4k	34k
		10	Qwen-7B	7.4k	19.4k
			GPT-20B	7.4k	29k
	Re-ranking	3	Qwen-3B	7.4k	296
			Qwen-7B	7.4k	18.4k
			Qwen-14B	7.4k	19.4k
			Autorefine	5.2k	7.3k
			GPT-20B	7.4k	21.9k
		5	Qwen-7B	7.4k	18.6k
			GPT-20B	7.4k	21.9k
		10	Qwen-7B	7.4k	17.8k
			GPT-20B	7.4k	19.9k
MSM	Retrieval	3	Qwen-3B	101k	6.6k
			Qwen-7B	101k	245k
			Autorefine	101k	58.2k
	Re-ranking	3	Qwen-3B	100.9k	6k
			Qwen-7B	100.8k	192.3k
			Autorefine	82k	56.9k
RQ	Retrieval	3	Qwen-3B	96.4k	4.9k
			Qwen-7B	96.3k	294.3k
			Autorefine	96.3k	41k

From Table 3, we note that our ASQ dataset contains a large number of traces spanning multiple agents, query sets, and retrieval configurations. As mentioned, the underlying query sets provide relevance judgements, while HQA also provides ground-truth answers. The scale of ASQ and the availability of these assessment resources allow it to support large-scale behavioural analyses and provide a basis for the future optimisation and evaluation of agent and retrieval configurations. Therefore, ASQ satisfies the *optimisability* and *assessability* properties. Finally, the incomplete-run rate does not exceed 0.68% for any evaluated configuration, ranging from 0% to 0.68%.

### Agentic trace statistics and query characteristics

To address RQ1, we now characterise the traces generated by the evaluated agents in terms of search-call volume, trace length, and intermediate-query length across query sets and retrieval settings.

From Table 3, we first note that the evaluated agents generate multiple retrieval requests per information need. In fact, the agents under study produce up to +401% (GPT-20B on HQA under the retrieval-only setting with *k* = 3) intermediate synthetic queries as compared to the original organic queries. In terms of relative increases in search calls across model sizes, we observe a sharp rise from Qwen-3B to Qwen-7B (up to +70%), followed by smaller increases when scaling to the larger Qwen-14B model (up to +3.8%). As these three variants belong to the same Search-R1 family and were fine-tuned using the same strategy [42], the results suggest that, within this specific setting, larger models generate more search calls. Similarly, when scaling from Qwen-14B to GPT-20B, we observe an increase in search calls up to +12%. However, in this last case the difference in search calls cannot be strictly attributable to model size alone, because they also differ in family and training procedure.

Table 4 complements the overview of ASQ, provided in Table 3, by reporting trace-length statistics and synthetic-query length statistics. From the trace-level statistics reported in Table 4, instead, we observe substantial variability in trace length across agents and configurations. Within the Search-R1 variants, which share the same generator family and training procedure and therefore provide a more controlled setting for comparison across model sizes, absolute standard deviation is higher for larger models, whereas relative standard deviation is higher for smaller ones; in other words, larger Search-R1 agents appear to generate more variable search-call volumes per input query, whereas smaller ones tend to search less and occasionally perform extensive searches. Across the other agent setups, the magnitude of trace-length variability remains configuration-dependent. From Table 4, we also note that trace length maxima are high: up to 186 on RQ, up to 77 on MSM, and up to 131 on HQA. This contrasts sharply with human search, as typically humans issue 2 or 3 queries per search session [12].

**Table 4:** Trace statistics for ASQ. For each corpus, search setting, *k* value and generator, we report statistics about trace length and query length. For traces generated from HQA, we also report the Exact-Match (EM) accuracy of the final answers as a percentage, with higher values indicating better accuracy.

Corpus	Search setting	Retrieval depth	Generator	Trace length		Query length	Answer accuracy
		*k*		Mean ± Std	Max	Mean ± Std	EM (%)
HQA	Retrieval	3	Qwen-3B	0.05±0.35	11	13.55±6.84	20.04
			Qwen-7B	1.66±1.43	9	8.32±4.52	44.66
			Autorefine	0.73±0.87	9	9.18±4.06	21.55
			GPT-20B	10.16±13.53	98	6.49±3.00	45.44
		5	Qwen-7B	1.56±1.33	8	8.43±4.58	45.23
			GPT-20B	10.24±14.98	131	6.55±3.05	47.27
		10	Qwen-7B	1.44±1.24	11	8.51±4.66	47.08
			GPT-20B	8.51±13.31	119	6.61±3.16	48.52
	Re-ranking	3	Qwen-3B	0.05±0.26	4	13.68±6.93	19.55
			Qwen-7B	1.36±1.16	7	8.51±4.95	54.72
			Qwen-14B	2.16±4.17	70	6.54±2.98	50.28
			Autorefine	0.71±0.84	6	9.36±4.16	24.40
			GPT-20B	6.34±10.69	91	6.99±3.27	51.33
		5	Qwen-7B	1.37±1.17	6	8.60±5.86	52.73
			GPT-20B	6.59±11.28	97	6.95±3.38	51.41
		10	Qwen-7B	1.30±1.10	8	8.66±7.31	52.46
			GPT-20B	5.58±10.45	119	6.95±3.44	51.99
MSM	Retrieval	3	Qwen-3B	0.10±0.47	17	7.00±3.00	—
			Qwen-7B	1.42±1.27	8	6.18±2.55	—
			Autorefine	0.45±0.89	54	6.39±2.49	—
	Re-ranking	3	Qwen-3B	0.08±0.38	11	6.88±2.70	—
			Qwen-7B	1.13±1.09	8	6.18±2.62	—
			Autorefine	0.43±1.17	77	6.36±2.50	—
RQ	Retrieval	3	Qwen-3B	0.52±6.58	186	7.61±4.68	—
			Qwen-7B	2.15±4.30	85	7.47±2.59	—
			Autorefine	0.32±0.52	16	7.91±2.57	—

From Table 4 we finally note that synthetic and organic queries have comparable lengths, except on HQA, where agents produced slightly longer queries. Among all the agents, Qwen-3B issues the longest queries.

To answer RQ1: search-call volume and trace length vary substantially across the evaluated agents, query sets, and retrieval settings. Agentic runs generate up to 401% additional intermediate queries, while individual trace lengths reach up to 186 search calls. Within the evaluated Search-R1 variants, larger models appear to generate more search calls. However, this relationship may be configuration-dependent and may not hold across other agent architectures. Finally, intermediate-query lengths are generally comparable to those of the original queries, except on HQA, where agents generate slightly longer queries.

#### Re-ranking, retrieval depth, and trace length.

We now address RQ2 by comparing, within each agent-corpus configuration, trace lengths for matched input queries when re-ranking is introduced or retrieval depth is increased.

At the aggregate level, Table 3 shows fewer search calls under re-ranking in several configurations, particularly for GPT-20B and Qwen-7B. Similarly, Table 4 reports lower mean trace lengths under re-ranking for these agents across the evaluated retrieval depths. However, these aggregate statistics do not capture the paired differences between configurations for individual input queries. To examine these differences, we compare trace lengths for matched input queries using the matched-pairs rank-biserial correlation, with 95% confidence intervals estimated from 1,000 paired bootstrap resamples. The complete results of this effect-size analysis are reported in S2 Table and show that the magnitude and direction of differences in trace lengths between the two retrieval settings vary substantially across agents, corpora, and retrieval depths; while re-ranking is associated with shorter traces in several configurations, the effect is negligible in others and can even reverse for specific agent-*k* combinations.

Similarly, to quantify the effect of retrieval depth on trace length, we performed paired rank-biserial correlation analyses across k∈{3,5,10}. The results of this additional effect-size analysis are reported in S3 Table and show that increasing *k* generally shortens traces, although the effect varies across agents and search settings.

Overall, our answer to RQ2 is that neither introducing re-ranking nor increasing retrieval depth produces a uniform change in trace length. Both are associated with shorter traces in several configurations, but the direction and magnitude of the differences depend on the agent and retrieval setting. Given this variability, it could be worth evaluating retrieval configurations on an agent- and task-specific basis.

### Agentic search behaviours and historical human-search patterns

We now turn to RQ3, which explores the similarities and differences between the agentic search behaviours observed in ASQ and those reported in historical human-search data, focusing on search-sequence length and query-reformulation patterns.

We begin our discussion by considering the length of the search sessions and agentic runs. From Table 4, we note that trace length maxima are high (up to 186 on RQ, up to 77 on MSM, and up to 131 on HQA). As mentioned, this contrasts sharply with human search. In fact, while agents can generate long traces, exposing retrieval systems to several requests for a single information need, humans are likely to abandon search sessions when unsatisfied — typically after 2 or 3 query reformulations [12]. As a result, agent traces can expose retrieval systems to longer sequences of reformulated queries than are typically observed in human search sessions. Overall, compared with human search, agentic search can generate substantially more retrieval requests per information need in the scenarios and settings considered. For tightly coupled agent–retrieval systems, our results motivate further investigation into agent-specific retrieval strategies, as the effects of retrieval-pipeline choices vary substantially across agents and configurations.

We next analyse how agents reformulate queries within these sequences, and compare them to those of humans. To do so, we adopt the methodology used in [13]. Specifically, we model aruns as Markov processes and we map each query $q_{i}$ in a trace to a state based on its relationship to its preceding queries in the same trace:

- IN: initial state, a new query *q*_0_ is issued.
- ADD: $q_{i}$ is an expanded version of $q_{i-1}$.
- REM: $q_{i}$ is a subsequence of $q_{i-1}$.
- REP: $q_{i}$ is a resubmission of $q_{i-1}$.
- DUP: $q_{i}$ is an exact duplicate of $q_{j}$ with j<(i−1).
- CH: $q_{i}$ results from any other reformulation strategy.
- OUT: terminal state (arun termination).

By aggregating all transitions between consecutive queries, for all traces, we build the *transition matrix*. Finally, for each non-terminal state *S*, we calculate the termination probability P(OUT|S), and insert it into the matrix.

Fig 1 shows the mean-aggregated transition matrices for agents across the different experimental setups. For comparison, we also show the transition matrix of human reformulations, with values taken from [13].

**Fig 1:**
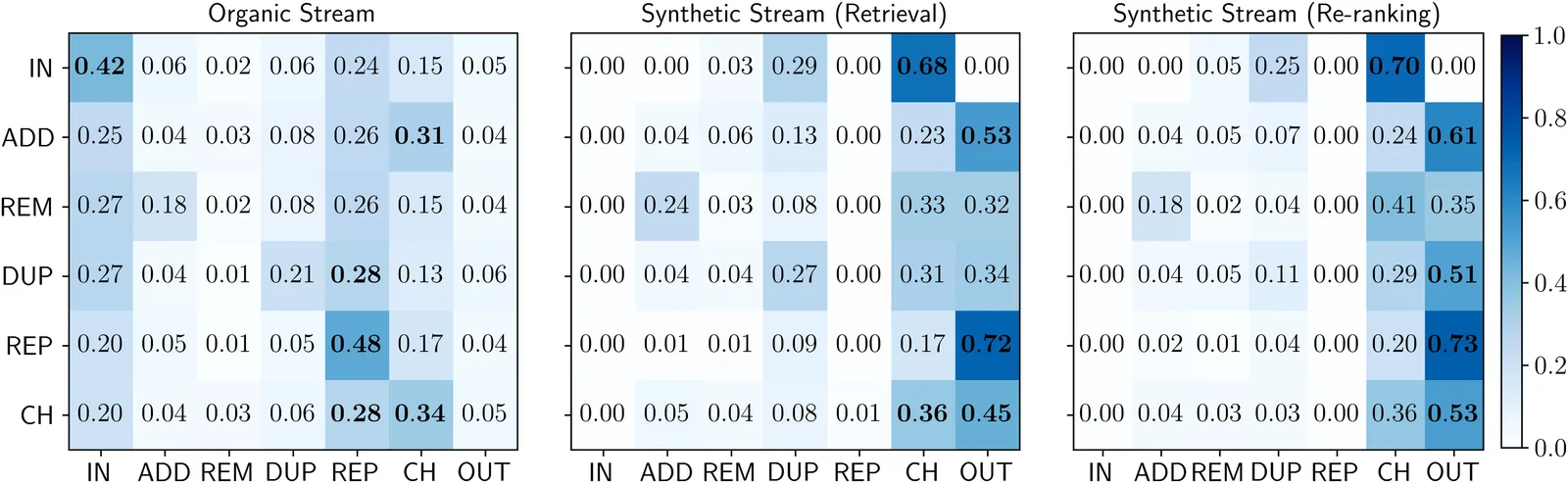
Transition matrices of human and agent query reformulations. The left matrix corresponds to the human query stream, whereas the centre and right matrices correspond to the synthetic query stream without and with re-ranking, respectively. Rows correspond to the current state and columns correspond to the next state.

Before analysing the matrices, we identify two structural differences that affect how some transition types should be interpreted. First, all reformulations in an arun are tied to the same initial query *q*_0_; consequently, the IN column is empty (all zeroes). For humans it is non-empty because multiple interleaved searches may collapse into the same session. Second, while REP in human sessions occurs by clicking on a “show more results” button, likely to increase recall, for aruns this occurs when $q_{i}=q_{i-1}$. Despite these structural differences, comparing query reformulation transition matrices remains informative from a retrieval-system perspective: in both settings the search system observes sequences of submitted queries that reveal how queries are refined, repeated, or reformulated over time.

We now compare the three matrices in Fig 1, focusing on differences between humans and agents. For humans, the most frequent arrival states are IN, REP, and CH, respectively, highlighting their multitasking, result browsing and substantial reformulation behaviours. Agents are more likely to transition to OUT, CH, and DUP, both under the retrieval and the re-ranking settings. The high OUT probability is partly explained by the construction of aruns, which are tied to a single initial query and terminate once the agent answers, stops, or fails. The high DUP probability indicates that agents often regress to previous queries. On the one hand, these DUP transitions may reflect the limited action space available to agents, which lack an explicit analogue of human result browsing. On the other hand, they can introduce redundant retrieval requests: under a fixed retrieval pipeline with deterministic ranking, re-issued queries are expected to retrieve the same results, potentially filling the agent context with duplicated evidence. The second behavioural difference is that agents favour CH and DUP over REP.

Finally, the transition matrices of the synthetic stream retrieval and re-ranking settings are highly similar. This suggests that agents rely on a learned reformulation strategy to generate queries, with limited adaptation to the retrieval setting at the level of the reformulation categories captured by our analysis.

To quantify the visual differences shown in Fig 1, in Table 5 we report the summary statistics of the Frobenius distances between the transition matrices of each configuration listed in Table 3 and the three fixed reference matrices shown in Fig 1, accounting for variability across traces using bootstrap resampling. For completeness, the configuration-specific Frobenius distances are reported in full in S1 Table.

**Table 5:** Frobenius distances between bootstrapped agent transition matrices and fixed reference transition matrices. We report the minimum, median, and maximum of the mean distances across all the bootstrapped agent transition matrices across all the configurations under study. For each configuration under study, we bootstrap traces 1,000 times. For each bootstrap sample, we compute the aggregate transition matrix and its Frobenius distance to each fixed reference matrix; we then average these distances across bootstrap samples.

Reference transition matrix	Min.	Median	Max.
Human	1.556	1.725	2.238
Synthetic (Retrieval)	0.352	1.125	1.753
Synthetic (Re-ranking)	0.459	1.079	1.686

From Table 5 we note that distances to the historical human reference are numerically larger than those to the aggregate agentic references. Given the experimental differences between the ASQ traces and the historical human reference, these distances provide a descriptive quantification of observed discrepancies rather than strong comparative evidence. They caution against transferring conclusions from the analysed human log directly to the evaluated agentic settings without additional validation.

Overall, our answer to RQ3 is that, in the settings considered, agents can generate substantially more search requests per information need than typically reported for human search. Agents and historical human sessions both frequently exhibit substantial query changes, but human sessions more often contain resubmissions of the immediately preceding query (REP), whereas agents more often return to an earlier query after one or more intervening reformulations (DUP). Note that the comparison should not be interpreted as a controlled human–agent comparison, since the underlying datasets and tasks, session definitions, and the semantics of some transition states differ between the two settings. However, our findings caution against treating historical human-search sessions as direct proxies for agentic workloads. In light of the longer search sequences and frequent query revisits observed in our evaluated settings, future work could investigate whether retrieval components such as repetition detectors, result reuse methods, and session-aware diversification are beneficial for agentic search.

### Within-trace dynamics

We next analyse how reformulation behaviour evolves over the course of aruns, aiming at answering RQ4.

In Figs 2 and 3, we respectively show the transition probabilities between pairs of consecutive iterations within the aruns generated by Qwen-7B and by GPT-20B on the HQA dataset. A comparison with human search sessions would be valuable, but to our knowledge, no public data currently allows such analysis.

**Fig 2:**
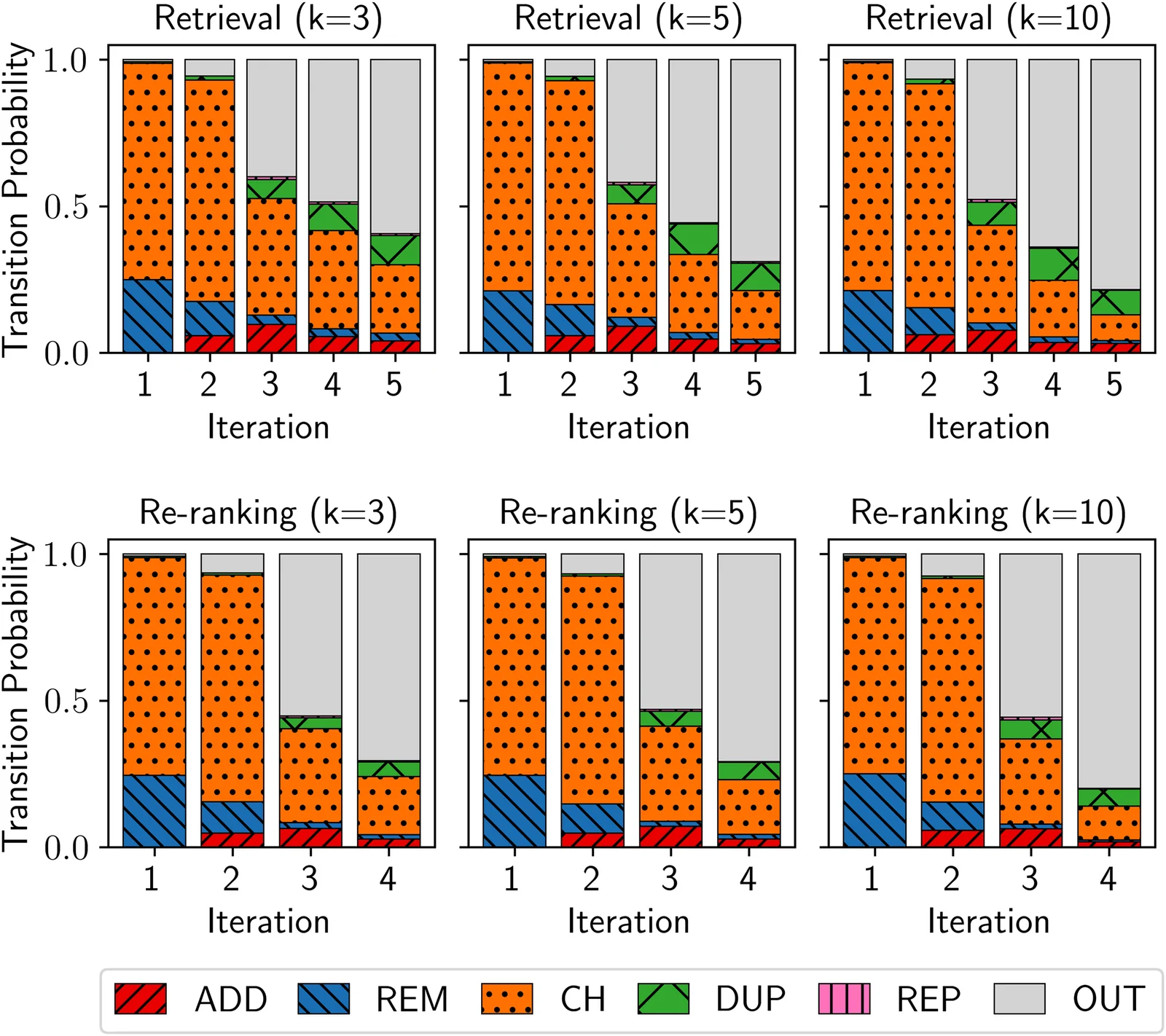
Transition probabilities across consecutive iterations for Qwen-7B on HQA. The top plot shows retrieval only, whereas the bottom plot shows retrieval followed by re-ranking. Stack *i* represents the transition probabilities from iteration (i−1) to *i*. The iteration range is truncated at the last iteration supported by at least 1,000 queries.

**Fig 3:**
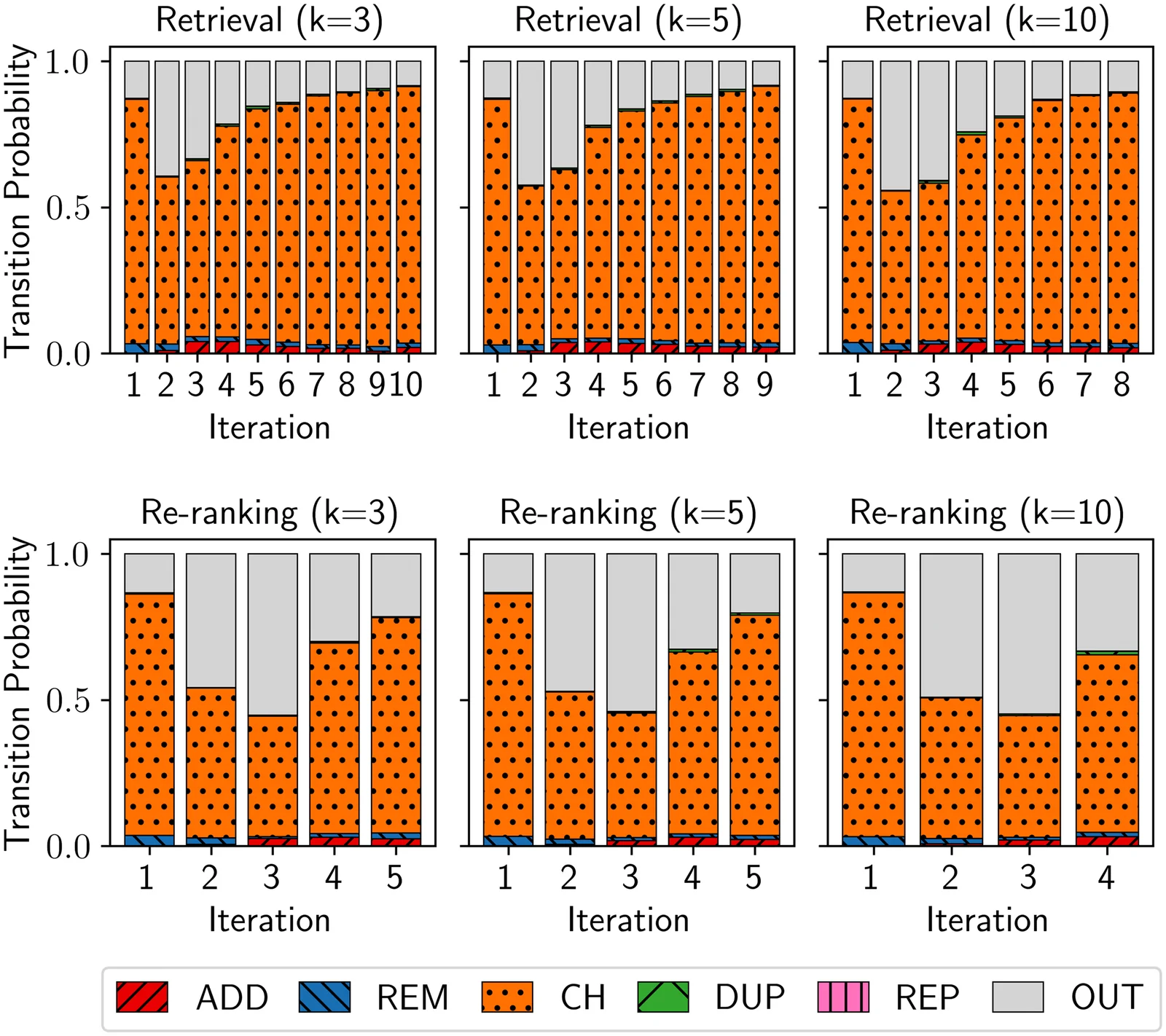
Transition probabilities across consecutive iterations for GPT-20B on HQA. The top plot shows retrieval only, whereas the bottom plot shows retrieval followed by re-ranking. Stack *i* represents the transition probabilities from iteration (i−1) to *i*. The iteration range is truncated at the last iteration supported by at least 1,000 queries.

Several behavioural patterns emerge from Figs 2 and 3. First, both agents rely heavily on substantial reformulations (CH) rather than on incremental term additions (ADD) and term removals (REM). Second, while after a few iterations Qwen-7B tends to resubmit prior queries (DUP) or to repeat the previous query (REP), GPT-20B continues to rely mostly on CH. Qwen-7B thus may adopt a trial-and-error approach where it cycles between trying to differentiate and reverting to old knowledge upon failure. Overall, these results suggest that repeated-query behaviour is more pronounced for Qwen-7B than GPT-20B. Third, both agents exhibit iteration-dependent differences in term-level reformulation. Term removal (REM) appears mainly in the early iterations, whereas term addition (ADD) becomes more visible from the third iteration onward. Overall, the choice of retrieval setting appears to affect iteration structure more strongly than the choice of *k*. Within retrieval-only or re-ranking settings, changing *k* does not substantially alter the dominant transitions and the aforementioned patterns.

For Qwen-7B, in Fig 4 we additionally report the transition probabilities across consecutive iterations separately by dataset for *k* = 3. This plot shows that Qwen-7B exhibits dataset-dependent transition patterns. As already observed in Fig 2, on HQA transitions are dominated by substantial reformulations (CH), with DUP transitions appearing mainly in later transitions and remaining comparatively rare. By contrast, on MSM and RQ, DUP transitions appear more frequently, particularly in the early iterations for MSM and in later iterations for RQ. Thus, for Qwen-7B DUP rates are lower on HQA than on the other two query sets. HQA is in-domain for Qwen-7B, whereas the other datasets are out-of-domain; this difference, however, cannot be attributed to domain alone, since the datasets also differ in several other characteristics, including query length, query type, and document characteristics. Nevertheless, it illustrates the value of analysing agentic traces across multiple datasets and motivates future controlled comparisons to disentangle these factors when evaluating agents and retrieval systems in agentic settings. Within the evaluated configurations in Fig 4, we also note that differences in DUP rates appear more pronounced across datasets than across retrieval settings. Moreover, the term-level reformulation patterns observed for Qwen-7B and GPT-20B in Figs 2 and 3 remain qualitatively similar across datasets. REM is concentrated in the early iterations, whereas ADD appears more frequently in later iterations. Finally, also in this case, the observed patterns are similar across the two search settings.

**Fig 4:**
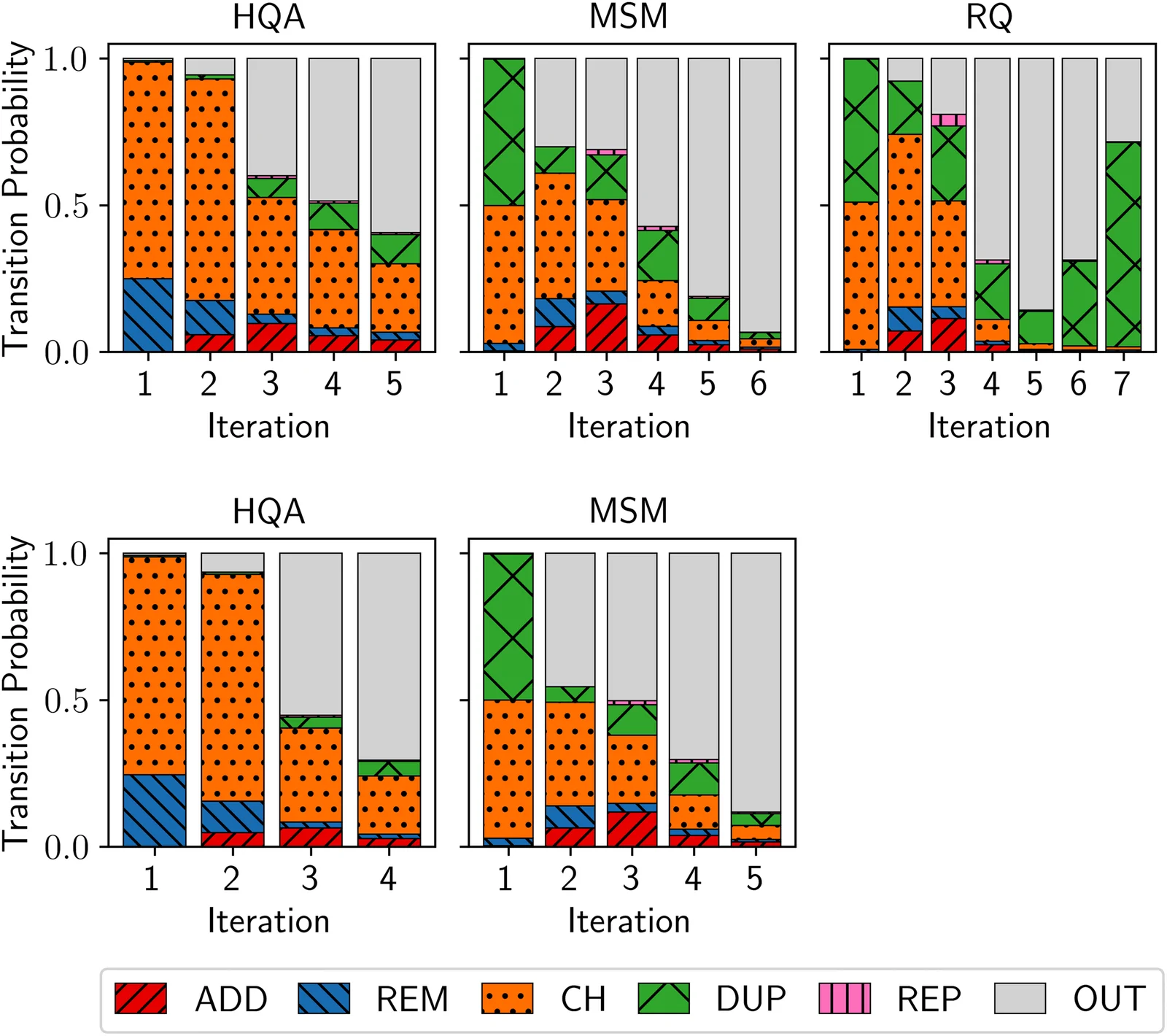
Transition probabilities across consecutive iterations for Qwen-7B on HQA, MSM, and RQ for *k* = 3. The top plot shows retrieval only, whereas the bottom plot shows retrieval followed by re-ranking for HQA and MSM only. No re-ranking panel is shown for RQ because re-ranking was not evaluated on this query set. Stack *i* represents the transition probabilities from iteration (i−1) to *i*. The iteration range is truncated at the last iteration supported by at least 1,000 queries.

Overall, our answer to RQ4 is that, for the two agents examined at the iteration level, query-reformulation patterns are iteration-dependent. Both agents rely mainly on CH; REM is more frequent early in the trace, whereas ADD becomes more frequent later. Qwen-7B exhibits more query repetition and revisiting than GPT-20B, with their timing varying across query sets. The dominant patterns remain broadly similar across the evaluated retrieval settings and depths.

### Trace behaviour and final-answer correctness

Having characterised agent search behaviour, we now turn to AQ1 to provide supplementary downstream context. Prior work suggests that long traces can arise when agents have difficulty converging to high-quality answers [58]. This motivates us to examine the relationship between trace length and final-answer correctness. Moreover, since our analysis shows that agents frequently repeat previously issued queries, we also explore the relationship between within-trace query repetition and answer correctness.

We report final-answer accuracy in terms of EM for HQA in Table 4. Our effect-size analysis, reported in S4 Table, shows that in most of the evaluated configurations, correctly answered queries are associated with shorter traces and lower within-trace query repetition than incorrectly answered ones. However, this pattern is not observed for every agent and retrieval configuration, and the effect sizes are generally small.

Overall, our answer to AQ1 is that shorter and less repetitive traces are generally associated with correct answers on HQA. This relationship is neither strong nor uniform, however, and varies across agents and retrieval configurations.

### ASQ: findings and opportunities

In this section, we presented an initial analysis of ASQ across the evaluated agents, query sets, and retrieval configurations. Our findings show that agents can produce long and repetitive query sequences; changes in re-ranking and retrieval depth do not uniformly reduce their length; and reformulation patterns vary across agents, query sets, and trace iterations. Moreover, aggregate reformulation patterns differ from those reported in historical human-search data, while the associations of trace length and query repetition with answer correctness are generally small and configuration-dependent. Overall, these analyses show how ASQ can support systematic studies of agent search behaviour across agent and retrieval configurations.

## Shortcomings

Our approach faces some challenges and limitations. As with all behavioural datasets, ASQ reflects the period in which it was collected. In a rapidly evolving field, this means it is inherently susceptible to becoming outdated soon. We mitigate this problem by releasing the code and documentation required to easily extend ASQ with new agents, retrieval systems, and benchmarks as the field evolves. Moreover, inference with agentic RAG systems is computationally intensive and requires substantial hardware resources, limiting the number of agents, retrieval settings, and query-set configurations that we could include. Nevertheless, the configurations included cover multiple agents, retrieval settings, and query types, enabling the study of a diverse range of agent search behaviours. Our study focuses on a limited set of open-source agents. This setup may limit how fully some observed behaviours generalise to proprietary systems or future model families. At the same time, this setting is practically relevant, as recent industry surveys and technical reports suggest that open-source and open-weight AI models are already widely used by enterprises [50,51]. Moreover, because full-scale production query logs of user queries submitted to agents are not publicly available, ASQ is grounded in benchmark query sets and does not capture important properties of real-world search activity beyond session-level behaviour, such as query frequency, temporal dynamics, user heterogeneity, or long-tail usage patterns. However, this setup provides a controlled setting for experimentation. Overall, these practical constraints limit the scope of ASQ, but do not undermine its purpose. Our goal is not to characterise the entire query stream of AI agents, but to identify some of the differences in search behaviours between humans and agents that retrieval systems may need to account for. Within this scope, the behaviours observed in ASQ, long search sessions, repeated queries, and reformulation patterns that differ from human search, are directly relevant to search systems. Finally, a limitation of our analysis is that the historical human-search data and the ASQ traces were collected under different tasks and conditions. However, a directly matched comparison was not possible with the available public data.

## Conclusions and future work

As AI agents are increasingly used in research, healthcare, education, and other settings, it becomes important to understand not only whether they produce plausible answers, but also how they search for supporting information. In this work, we release ASQ, a dataset capturing how open-source agentic RAG systems search. Grounded in three diverse query sets, ASQ enables the analysis of how these systems reformulate queries, retrieve information, and interact with retrieval systems over multiple steps. Since the query sets we ground ASQ on provide relevance judgements and, in some cases, ground-truth answers, ASQ provides trace-level data that can support the future evaluation and optimisation of both retrieval systems and agents. Our results show that the evaluated agentic traces differ from the reported historical human-search patterns in within-session query volume, trace length, repetition, and reformulation dynamics. We observe recurrent patterns including repeated reformulations, redundant queries, and substantial variation in trace lengths. These patterns motivate future evaluation of whether, and under which conditions, retrieval components should be adapted for agentic search. However, our findings are based on controlled, benchmark-based ASQ traces generated by open-source agents and may therefore not fully capture proprietary systems or real-world production query logs. Future work could quantify run-to-run variability through repeated, multi-seed generation, compare humans and agents under matched conditions, investigate retrieval effectiveness, latency, and cost, and could define concrete effectiveness-efficiency evaluation protocols that relate trace-level behaviours to system efficiency. Finally, if such data become publicly available, future work could analyse production traces that include complete agent, task, and trace context.

## Supporting information

S1 Table Complete comparison of Frobenius distances between configuration-specific transition matrices and aggregate transition matrices of the synthetic and organic streams. (PDF)

S2 Table Effect-size analysis of the impact of re-ranking on agent trace length. (PDF)

S3 Table Effect-size analysis of the impact of retrieval depth on agent trace length. (PDF)

S4 Table Effect-size analysis of trace length and query repetition rate between agentic traces that produced correct and incorrect answers. (PDF)
