# Supplementary material for: How AI agents search
Source: PLoS One. 2026 Oct 21;21(10):e0360157. doi: 10.1371/journal.pone.0360157 (PMC13686499; doi:10.1371/journal.pone.0360157)
Supplement: Supplementary file 1 [file pone.0360157.s001.pdf]

S1 Table. Frobenius distance between agent and reference transition matrices. For each corpus, search setting, retrieval depth k, and generator, we compare the agent transition matrix with three reference matrices: the human matrix, the aggregate retrieval matrix (agentic), and the aggregate re-ranking matrix (agentic). Similarity is measured using the Frobenius distance. Lower values indicate that the agent's transition behaviour is more similar to the corresponding reference. For each row, the smallest mean distance across the three references is highlighted. Values in square brackets are 95% confidence intervals estimated using bootstrap resampling.

				Distance to reference matrix		
Corpus	Search setting	Retrieval depth k	Generator	Human reference	Aggregate retrieval reference	Aggregate re-ranking reference
HQA	Retrieval	3	Qwen-3B	$2.16_{[1.93,2.28]}$	$1.75_{[1.66,1.86]}$	$1.69_{[1.59,1.79]}$
			Autorefine	$2.10_{[2.03,2.14]}$	$1.02_{[0.96,1.07]}$	$0.84_{[0.79,0.89]}$
			Qwen-7B	$1.57_{[1.55,1.59]}$	$0.76_{[0.70,0.84]}$	$0.69_{[0.63,0.77]}$
			GPT-20B	$1.70_{[1.64,1.78]}$	$1.40_{[1.27,1.52]}$	$1.42_{[1.29,1.54]}$
	Re-ranking	3	Qwen-3B	$2.24_{[1.91,2.44]}$	$1.65_{[1.52,1.83]}$	$1.57_{[1.42,1.77]}$
			Autorefine	$2.00_{[1.92,2.09]}$	$0.93_{[0.87,1.00]}$	$0.75_{[0.69,0.82]}$
			Qwen-7B	$1.73_{[1.69,1.77]}$	$0.72_{[0.68,0.77]}$	$0.56_{[0.52,0.62]}$
			Qwen-14B	$1.65_{[1.62,1.68]}$	$1.00_{[0.92,1.09]}$	$1.03_{[0.94,1.13]}$
			GPT-20B	$1.67_{[1.59,1.78]}$	$1.33_{[1.24,1.44]}$	$1.32_{[1.24,1.42]}$
		5	Qwen-7B	$1.70_{[1.67,1.73]}$	$0.77_{[0.71,0.85]}$	$0.63_{[0.56,0.71]}$
			GPT-20B	$1.65_{[1.61,1.71]}$	$1.21_{[1.05,1.36]}$	$1.22_{[1.05,1.37]}$
		10	Qwen-7B	$1.81_{[1.76,1.85]}$	$0.73_{[0.71,0.76]}$	$0.56_{[0.54,0.59]}$
			GPT-20B	$1.65_{[1.60,1.75]}$	$1.13_{[0.91,1.41]}$	$1.15_{[0.93,1.41]}$
MSM	Retrieval	3	Qwen-3B	$1.93_{[1.86,2.01]}$	$1.30_{[1.27,1.34]}$	$1.26_{[1.24,1.30]}$
			Qwen-7B	$1.56_{[1.55,1.57]}$	$0.37_{[0.37,0.38]}$	$0.49_{[0.49,0.50]}$
			Autorefine	$2.06_{[2.02,2.10]}$	$1.12_{[1.08,1.15]}$	$1.02_{[0.99,1.07]}$
	Re-ranking	3	Qwen-3B	$2.04_{[1.95,2.14]}$	$1.40_{[1.35,1.46]}$	$1.34_{[1.29,1.40]}$
			Qwen-7B	$1.71_{[1.70,1.72]}$	$0.47_{[0.47,0.48]}$	$0.46_{[0.45,0.47]}$
			Autorefine	$2.17_{[2.11,2.23]}$	$1.23_{[1.19,1.27]}$	$1.13_{[1.09,1.16]}$
RQ	Retrieval	3	Qwen-3B	$2.01_{[1.92,2.15]}$	$1.52_{[1.43,1.69]}$	$1.57_{[1.47,1.74]}$
			Qwen-7B	$1.56_{[1.55,1.56]}$	$0.35_{[0.35,0.36]}$	$0.58_{[0.57,0.58]}$
			Autorefine	$2.16_{[2.10,2.28]}$	$1.37_{[1.27,1.56]}$	$1.26_{[1.15,1.48]}$
