# Supplementary material for: How AI agents search
Source: PLoS One. 2026 Oct 21;21(10):e0360157. doi: 10.1371/journal.pone.0360157 (PMC13686499; doi:10.1371/journal.pone.0360157)
Supplement: Supplementary file 3 [file pone.0360157.s003.pdf]

S3 Table. Effect of re-ranking on trace length. This analysis tests whether adding a re-ranking stage shortens agent traces compared with retrieval-only search for the same query. For each corpus, retrieval depth k, and generator, we report the matched-pairs rank-biserial correlation, r_{rb}, which summarizes the direction and size of the difference between the two settings. Positive values indicate shorter traces with re-ranking, negative values indicate longer traces with re-ranking, and values close to zero indicate little difference. To show how many observations support each estimate, and how many comparisons are informative for r_{rb}, we also respectively report the number of paired traces compared and the number of pairs with different trace lengths. The values reported in square brackets are 95% confidence intervals, estimated from 1k paired bootstrap resamples; they indicate the uncertainty around r_{rb}. Cells highlighted in red indicate negative effect sizes, whereas those highlighted in green indicate positive effect sizes; light green denotes positive estimates below 0.5, while dark green denotes estimates of 0.5 or above. Cells without highlighting indicate that their confidence intervals include 0 and, therefore, that the direction of the effect is not clearly established.

Corpus	Retrieval depth k	Generator	Paired traces compared	Pairs with different trace lengths	Effect of re-ranking r_{rb} [95% CI]
HQA	3	Qwen-3B	7.39k	260	$-0.06_{[-0.19,0.08]}$
		Qwen-7B	7.35k	3.88k	$+0.57_{[0.54,0.59]}$
		Autorefine	6.82k	3.12k	$+0.04_{[-0.00,0.07]}$
		GPT-20B	7.16k	6.12k	$+0.69_{[0.67,0.71]}$
	5	Qwen-7B	7.35k	3.59k	$+0.44_{[0.41,0.47]}$
		GPT-20B	7.17k	6.01k	$-0.14_{[-0.17,-0.11]}$
	10	Qwen-7B	7.35k	3.27k	$+0.37_{[0.34,0.40]}$
		GPT-20B	7.16k	3.94k	$+0.35_{[0.32,0.38]}$
MSM	3	Qwen-3B	101k	4.3k	$+0.09_{[0.05,0.12]}$
		Qwen-7B	101k	57k	$+0.61_{[0.61,0.62]}$
		Autorefine	90k	26k	$+0.05_{[0.03,0.06]}$
