# Supplementary material for: How AI agents search
Source: PLoS One. 2026 Oct 21;21(10):e0360157. doi: 10.1371/journal.pone.0360157 (PMC13686499; doi:10.1371/journal.pone.0360157)
Supplement: Supplementary file 4 [file pone.0360157.s004.pdf]

S4 Table. Effect sizes for associations between trace length and within-trace query repetition and answer correctness HQA. For each agent, k value, and search setting, we compare trace length and query repetition between queries answered correctly and incorrectly by the agents. We report the number of correct and incorrect answers, and the independent-samples rank-biserial correlation r_{rb} with 95% confidence intervals estimated from 1k stratified bootstrap resamples. Negative r_{rb} values indicate that correct answers tend to be associated with lower trace lengths or lower query repetition rates, whereas positive values indicate higher values. Cells highlighted in red indicate negative effect sizes, whereas those highlighted in green indicate positive effect sizes. Cells without highlighting indicate that their confidence intervals include 0 and, therefore, that the direction of the effect is not clearly established.

Search setting	k	Generator	Correct answers	Incorrect answers	Trace length r_{rb} [95% CI]	Query repetition rate r_{rb} [95% CI]
Retrieval	3	Qwen-3B	1.48k	5.92k	$+0.02_{[+0.01,+0.03]}$	$-0.00_{[-0.00,+0.00]}$
		Qwen-7B	3.31k	4.10k	$-0.24_{[-0.26,-0.21]}$	$-0.12_{[-0.13,-0.10]}$
		Autorefine	1.60k	5.81k	$+0.02_{[-0.02,+0.05]}$	$+0.02_{[+0.01,+0.04]}$
		GPT-20B	3.37k	4.04k	$-0.19_{[-0.21,-0.16]}$	$-0.11_{[-0.13,-0.10]}$
	5	Qwen-7B	3.35k	4.06k	$-0.19_{[-0.22,-0.17]}$	$-0.10_{[-0.12,-0.09]}$
		GPT-20B	3.50k	3.91k	$-0.15_{[-0.18,-0.13]}$	$-0.10_{[-0.11,-0.09]}$
	10	Qwen-7B	3.49k	3.92k	$-0.17_{[-0.19,-0.14]}$	$-0.10_{[-0.11,-0.08]}$
		GPT-20B	3.59k	3.81k	$-0.14_{[-0.16,-0.12]}$	$-0.10_{[-0.11,-0.08]}$
Re-ranking	3	Qwen-3B	1.45k	5.96k	$+0.03_{[+0.01,+0.04]}$	$+0.00_{[-0.00,+0.00]}$
		Qwen-7B	4.05k	3.35k	$-0.11_{[-0.14,-0.09]}$	$-0.05_{[-0.06,-0.04]}$
		Qwen-14B	3.72k	3.68k	$-0.13_{[-0.15,-0.10]}$	$-0.03_{[-0.05,-0.02]}$
		Autorefine	1.81k	5.60k	$+0.07_{[+0.04,+0.10]}$	$+0.01_{[+0.00,+0.02]}$
		GPT-20B	3.80k	3.60k	$-0.09_{[-0.11,-0.06]}$	$-0.06_{[-0.07,-0.05]}$
	5	Qwen-7B	3.91k	3.50k	$-0.13_{[-0.15,-0.10]}$	$-0.06_{[-0.07,-0.05]}$
		GPT-20B	3.81k	3.60k	$-0.06_{[-0.09,-0.04]}$	$-0.06_{[-0.07,-0.05]}$
	10	Qwen-7B	3.89k	3.52k	$-0.12_{[-0.14,-0.09]}$	$-0.05_{[-0.06,-0.04]}$
		GPT-20B	3.85k	3.56k	$-0.07_{[-0.09,-0.04]}$	$-0.06_{[-0.07,-0.05]}$
